# MDCT in the Differentiation of Adrenal Masses: Comparison between Different Scan Delays for the Evaluation of Intralesional Washout

**DOI:** 10.1155/2013/957680

**Published:** 2013-03-07

**Authors:** Giuseppe Angelelli, Maria Elisabetta Mancini, Marco Moschetta, Pasquale Pedote, Pasquale Pignataro, Arnaldo Scardapane

**Affiliations:** D.I.M. Interdisciplinary Department of Medicine, Aldo Moro University of Bari Medical School, Piazza Giulio Cesare 11, 70124 Bari, Italy

## Abstract

*Purpose*. To evaluate the accuracy of the washout in the differential diagnosis between adenomas and nonadenomas and to compare the obtained results in delayed CT scans at 5, 10 and 15 minutes. *Methods*. Fifty patients with adrenal masses were prospectively evaluated. CT scans were performed by using a 320-row MDCT device, before and after injection of contrast material. In 25 cases, delayed scans were performed at 5′ and 10′ (group 1), while in the remaining 25, at 5′ and 15′ (group 2). Absolute and relative wash-out percentage values (APW and RPW) were calculated. *Results*. Differential diagnosis between adenomas and nonadenomas was obtained in 48/50 (96%) cases, with sensitivity, specificity, and accuracy values of 96%, 95%, and 96%, respectively. In group 1, APW and RPW values were, respectively, 69.8% and 67.2% at 5′ and 75.9% and 73.5% at 10′ for adenomas and 25.1% and 15.8% at 5′ and 33.5% and 20.5% at 10′ for nonadenomas. In group 2, APW and RPW values were 63% and 54.6% at 5′ and 73.8% and 65.5% at 15′ for adenomas and 22% and 12.5% at 5′ and 35.5% and 19.9% at 15′ for nonadenomas. *Conclusions*. The evaluation of the wash-out values in CT scans performed at 5′, 10′, and 15′ provides comparable diagnostic results. CT scans performed at 5′ are, therefore, to be preferred, since they reduce the examination time and patient discomfort.

## 1. Introduction

In recent years, the detection of adrenal expansive lesions during CT examinations has become common, even in patients without endocrinological symptoms, because of the increasing number of investigations carried out for different clinical problems, with a prevalence varying from 0.35% to 9% in different series [1, 2].

After recognizing an expansive adrenal lesion, the differentiation between adenomas and nonadenomas becomes crucial for patient's prognosis and for the choice of the therapeutic approach [3–6].

The role of CT for differential diagnosis has been studied in numerous investigations, and the accuracy of CT scans before and after injection of contrast material has been reported, even using dual energy CT scanners [7].

In case of unenhanced CT scans, intralesional density values of less than 10 HU indicate an adenoma with high accuracy. In contrast, intralesional density values greater than 10 HU are more common in nonadenomas, but they cannot exclude the possibility of adenomas with low-intra-cytoplasmic fat content [8–11].

CT scans after injection of contrast material mainly offer the evaluation of the peak density and intralesional washout for differential diagnosis between adenomas and nonadenomas. There is no unanimous agreement in the literature for the optimal scan delay to evaluate this parameter; according to some authors, the optimal delay is represented by 10 minutes after intravenous injection of contrast material, to others 15 minutes, and according to other experiences, earlier CT scans performed at 5 minutes can be used in this field [12–21]. 

The purpose of this study is to evaluate the accuracy of the wash-out in the differential diagnosis between adenomas and nonadenomas and to compare the results obtained in CT scans performed at 5, 10, and 15 minutes after intravenous injection of contrast material.

## 2. Materials and Methods

Between February 2009 and December 2011, 50 subjects (26 males and 24 females) being diagnosed with adrenal lesions from 1 to 12 cm in diameter were prospectively studied with CT. All fluid cyst-like lesions or lesions with fat content related to myelolipomas were excluded from our series.

In 13 cases, CT examination was performed for the presence of significant endocrinological symptoms, in 8 cases, after incidental ultrasound finding of adrenal mass, and in 29 cases, the diagnosis was occasional during abdominal CT examinations performed for various clinical problems.

Patients were aged between 35 and 83 years (mean age 59). Patients were randomly assigned to one of the following two groups, based on the type of late scans: group 1 (scans performed at 5′ and 10′) and group 2 (scans performed at 5′ and 15′).

The lesions recognized in the first group were represented by 10 adenomas, 4 adrenal carcinomas, 1 ganglioneuroma, 4 pheochromocytomas, and 6 metastases (4 lung carcinomas, 1 renal cell carcinoma, and 1 ovarian carcinoma), in the second group, 12 adenomas, 3 adrenal carcinomas, 3 pheochromocytomas and 7 metastases (4 lung carcinomas, 2 colon carcinomas, and 1 melanoma).

The diagnosis was histologically confirmed in 21 cases (7 adrenal carcinomas, 1 ganglioneuroma, 1 metastasis, 7 benign pheochromocytomas, and 5 adenomas); in the remaining patients, it was based on the densitometric appearance and clinical-radiological evolution of the disease, as assessed by CT twice a year. The mean followup was 1.5 years. In particular, the absence of morphological and volumetric variations of the lesion in the subsequent controls was considered significant of adenoma, while its size increase was considered indicative of metastasis.

CT examinations were performed by using a 320-row multidetector CT system (Aquilion One, Toshiba Medical Systems, Otawara, Japan), and the following acquisition parameters were used: slice thickness 0.5 mm, and increment 0.5 mm, rotation time 0.5 s; 120/200 kVp/mAs. An automatic dose modulation system was used in all cases.

In all cases, images were acquired before and after intravenous injection of contrast material (Iomeron 400 Bracco, Milan, Italy), injected in a quantity equal to 1.5 mL per kg of body weight, up to a maximum of 120 mL, at a flow rate of 3.5 mL per second. Scans were performed in the portal venous phase (50–60 seconds after the injection of contrast material) and in the late phases, carried out, as already reported, in 25 patients at 5 and 10 minutes and in 25 patients at 5 and 15 minutes.

The obtained data were transferred and analyzed on a workstation (HP XW 8600) equipped with a software dedicated to image reconstruction (Vitrea FX 2.1, Vital Images, Minneapolis, MN, USA). Axial and reconstructed images were analyzed by two independent blinded radiologists with 30 and 6 years of experience in abdominal CT, and the following parameters were considered:

- densitometry of the lesion in unenhanced scans;
- densitometry after contrast material injection, assessed by applying a large round or oval region of interest (ROI) excluding any calcification, areas of necrosis, or cystic degeneration;
- absolute intralesional percentage wash-out (APW) in scans at 5′, 10′, and 15′;
- relative intralesional percentage wash-out (RPW) in scans at 5′, 10′, and 15′.

In order to calculate the APW and RPW values, the following formulas were used, respectively: APW = 100 × ([EA − DA]/[EA − PA]); RPW = 100 × ([EA − DA]/[EA]), where EA = early-phase postcontrast attenuation; DA = delayed-phase postcontrast attenuation; PA = precontrast attenuation. An APW of more than 60% and an RPW of more than 40% were considered significant for adenoma, independently from the used scan delay [12].

Analysis of our sample was performed by using descriptive statistics and examining precontrast and postcontrast density of the lesions and APW and RPW values. The sensitivity, specificity, and diagnostic accuracy values were obtained. The degree of concordance between the two readers was assessed by using Cohen's Kappa statistics: poor concordance (*k* < 0.01), low concordance (*k* = 0.01 to 0.20), fair agreement (*k* = 0.21 to 0.40), good agreement (*k* = 0.41–0.60), substantial agreement (*k* = 0.61–0.80), and almost perfect agreement (*k* = 0.81–1.00).

## 3. Results 

Mean densitometric values for each type of lesion are reported in Tables 1 and 2. The densitometric values of the adenomas in unenhanced scans ranged between −6 and 19.4 HU (mean 4.6 HU; standard deviation 0.9). In particular, density values of less than 10 HU were found in 16 out of 22 (72.7%) cases; values of more than 10 HU were found in 6 out of 22 (27.3) cases.

Nonadenomas presented an unenhanced density from between 14 and 43.9 HU (mean 29.5 HU; standard deviation 9.2).

After intravenous injection of contrast material, in the portal venous phase, adenomas showed a mean enhancement of 77.5 HU (range 38.2 to 132.4 HU), while nonadenomatous lesions a mean value of 75.4 HU (range 38.2–188.1 HU).

**Figure 1:**
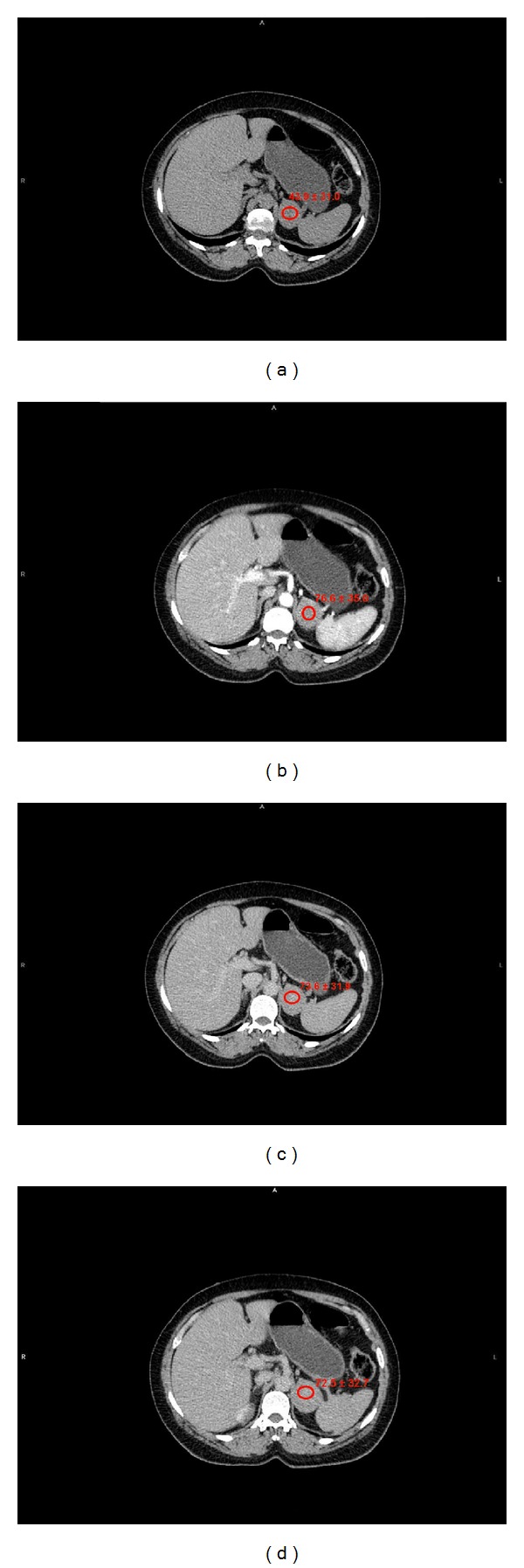
Pheochromocytoma. (a) Unenhanced attenuation values: 43,9 ± 41 HU. (b) Portal-phase attenuation values: 76,6 ± 35 HU. (c) 5′ attenuation values: 73,6 ± 31,9 HU. (d) 10′ attenuation values: 72,5 ± 32,7 HU. Absolute percentage wash-out at 5′ : 9%. Relative percentage wash-out at 5′ : 3,9%. Absolute percentage wash-out at 10′ : 12,5%. Relative percentage wash-out at 10′ : 5,3%.

Among the group of patients studied at 5 and 10 minutes, in the scans at 5′, the mean values of APW for adenomas ranged between 61% and 79.2% (mean: 69.8%) and in the scans at 10′, between 72.7% and 80.7% (mean: 75.9%). Nonadenomas ranged between 0.7% and 60.3% (mean: 25.1%) at 5′ and between 10.5% and 70.5% (mean: 33.5%) at 10′ (Figure 1). The values of RPW for adenomas ranged between 57.9% and 78.8% (mean 67.2%) at 5′ and between 62.9% and 82.1% (mean 73.5%) at 10′. For nonadenomas, RPW ranged between 0.4% and 52.5% (mean 15.8%) at 5′ and between 5.3% and 61.4% (mean 20.5%) at 10′.

**Figure 2:**
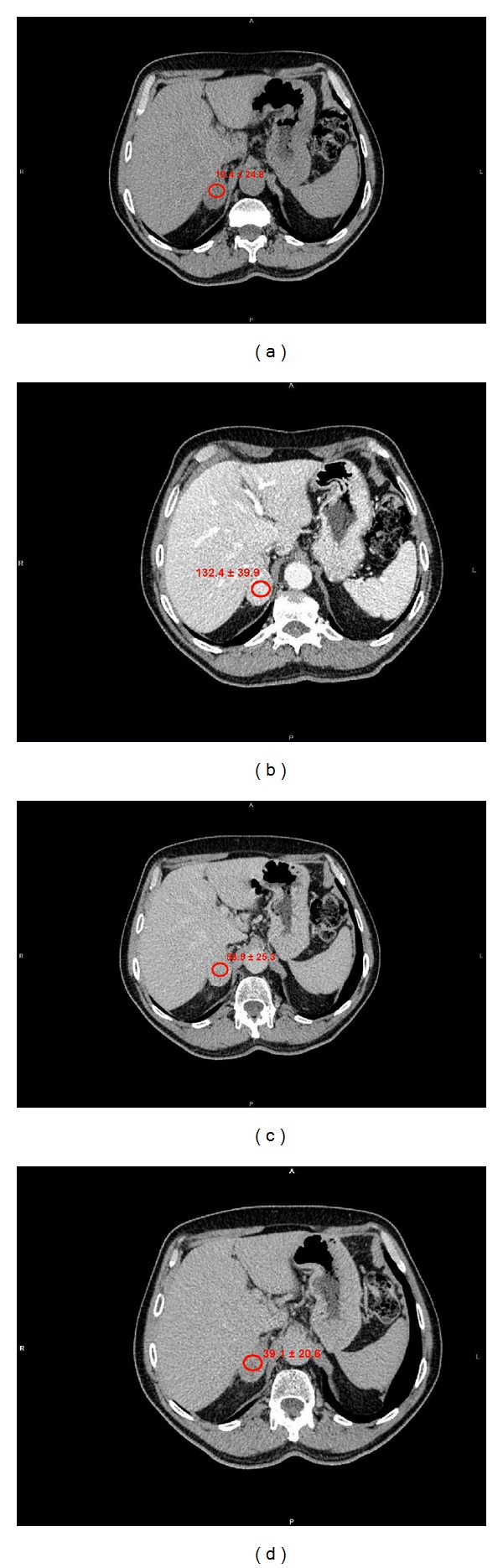
Lipid-poor adenoma. (a) Unenhanced attenuation values: 19,4 ± 24,8 HU. (b) Portal-phase attenuation values: 132,4 ± 39,9 HU. (c) 5 minutes attenuation values: 58,5 ± 25,3 HU. (d) 15 minutes attenuation values: 39,1 ± 20,6 HU. Absolute percentage wash-out at 5′ : 65,3%. Relative percentage wash-out at 5′ : 55,8%. Absolute percentage wash-out at 15′ : 82,5%. Relative percentage wash-out at 15′ : 70,4%.

Among the group of patients studied at 5 and 15 minutes, in the scans at 5′, values between 35.6% and 82.4% (mean 63%) and 31.3% and 80.6% (mean 54.6%) emerged for APW and RPW, respectively, in case of adenomas. In 15′ scans, APW values ranged between 44.2% and 91.4% (mean 73.8%) and RPW values between 39.4% and 83.3% (mean 65.5%) (Figure 2). For nonadenomas, APW and RPW values at 5′ varied, respectively, between 11.9% and 33% (mean 22%) and between 5% and 20.9% (mean 12.5%). At 15′, values ranged between 22.9% and 42.2% (mean 35.5%) and between 12.7% and 31.8% (mean 19.9%). The degree of concordance between the two readers was almost perfect(*k*  =  0.82).

Basing on the wash-out of the examined lesions, a differential diagnosis between adenomas and nonadenomas was obtained in 48/50 (96%) cases, with sensitivity, specificity, and diagnostic accuracy values of 96%, 95%, and 96%, respectively. In particular, APW and RPW provided comparable results, and densitometric values obtained by using different scan delays did not cause significant diagnostic changes. 

Among the two cases of incorrect diagnosis, one was the case of an adrenal metastasis from renal cell carcinoma in which the obtained wash-out values were significant for adenoma. In particular, APW values of 60.3% at 5′ and 70.5% at 10′ were obtained in this patient, and 52.5% at 5′ and 61.4% at 10′ were found for RPW. The second case was represented by a lipid-poor adenoma in which the obtained wash-out values were significant for nonadenoma. In particular, APW was 35.6% at 5′ and 44.2% at 15′, while the RPW was 31.3% at 5′ and 39.4% at 15′. 

## 4. Discussion 

The detection of adrenal expansive lesions during CT examination is frequent, with a prevalence varying between 0.35% and 9% in different series [1, 2]. In 50%–80% of cases, they are represented by adenomas, whereas nonadenomas are most often represented by metastases, adrenal carcinomas (<5%), pheochromocytomas (5%), myelolipomas (5%–10%), and cysts (1%–5%) [22–24]. The metastases originate more often from carcinomas of the lung, breast, kidney, thyroid, colon, and melanoma and represent 20%–50% of adrenal masses diagnosed in patients with known neoplastic disease [3, 22, 25]. The differential diagnosis between adenomas and nonadenomas with imaging techniques is of particular importance for an adequate prognostic and therapeutic approach, being able to avoid the use of invasive procedures such as biopsy or unnecessary prolonged followup.

In the differentiation between adenomas and nonadenomas, morphological, histological, and physiological criteria are usually used.

The morphological criteria considering the size and the homogeneous or inhomogeneous appearance of the lesion provide useful elements for differential diagnosis between adenomas and nonadenomas but need to be always combined with other parameters. In particular, adenomas are most often lesions with regular margins, small in size, with a mean value of less than 3 cm, and have a homogeneous density. In autopsy series, only 2% of adrenal adenomas had a diameter greater than 4 cm and 0.03% over 6 cm. Metastases, carcinomas, and pheochromocytomas, on the contrary, have more frequently a diameter larger than 4 cm, irregular contours, and an inhomogeneous appearance for the presence of areas of necrosis, hemorrhage, and intralesional cystic degeneration [3, 5, 6, 23, 24, 26].

The histological criteria are based on the evaluation of intracellular lipids within the adrenal lesion. About 70% of adrenal adenomas, in fact, are made up of cells containing intracytoplasmic lipid deposits, which represent the precursors of their secreted hormone and confer a low density to the mass in the unenhanced CT scans. In particular, as reported in the literature, an intralesional density value of less than 0 UH in unenhanced scans allows the diagnosis of adenoma with a sensitivity of 47% and a specificity of 100% [8]. A sensitivity of 71%–79% and specificity of 96%–98% are instead reported with a threshold value of 10 UH, usually considered in clinical practice [10, 17, 22, 27]. As reported by Stadler et al., slice thickness of ≤3 mm and low *Kv * values are indicated for evaluating the densitometric values of adrenal lesions in order to optimize the obtained results [28].

In our experience, the unenhanced scans were significant for the differential diagnosis between adenomas and nonadenomas in 75% of cases, and these data are substantially similar to those reported in the literature. In no case of nonadenomas, a basal density value of less than 10 HU was found. 

The morphological criteria represent, therefore, an important parameter of evaluation but have some limitations.

- They do not allow a diagnostic orientation in case of lipid-poor adenomas (approximately 30% of cases), which have a density greater than 10 HU.
- Unenhanced CT scans are often not used in the followup of cancer, and therefore the histological criteria cannot be evaluated.
- The possibility exists that an adrenal carcinoma contains foci of intracytoplasmic lipids [25], as well as exceptionally metastatic from clear cell renal carcinoma and hepatocellular carcinoma [3].

The physiological criteria are represented by the vascular enhancement and the washout of the lesion.

According to some authors, in the post-contrast scans performed in the portal venous phase, nonadenomas have an average lower density than in adenomas [14]. For others, however, these scans do not provide any element of differential diagnosis, because both adenomas and nonadenomas show a significant enhancement in the early phase, with substantially overlapped density values [12, 15, 16, 23]. These data also emerged in our experience, in which an average density of 77.5 HU was found in adenomas, and a mean value of 75.4 HU in case of nonadenomas.

Regarding the intralesional wash-out, in 1989, Krestin et al. evaluated 38 adrenal masses by using MRI with Gd-DTPA and firstly emphasized that adenomas and nonadenomas could be differentiated on this basis, highlighting a more rapid wash-out of contrast material in case of adenomas compared with pheochromocytomasand malignancies,which tend to retain the contrast material for a longer period [26]. Numerous studies have subsequently emphasized the role of the analysis of intralesional washout in late CT scans after intravenous injection of contrast material, although there is no agreement yet on the timing of image acquisition and the values of wash-out to be considered significant for the differential diagnosis.

Some authors have evaluated the contribution of the late scans performed at 15′ after the injection of contrast material, reporting a sensitivity and specificity of 79%–89% and 92%–96% for APW values of more than 60% and a sensitivity and a specificity of 82%–83% and 92%–93% for RPW values of more than 40% [14–17]. Other researchers experienced the use of scans at 10 and 5 minutes in order to obtain a simplification and a reduction of execution times for CT examinations. Blake et al. evaluated 122 adrenal masses by using a protocol that included CT scans in 10′, with a threshold value of 52% for the APW and 37.5% for the RPW, and reported a sensitivity and specificity of 100% and 98%, respectively [18]. In a series of 323 adrenal lesions, Sangwaiya et al., always using a delay of 10′ and considering different threshold values for APW and RPW, obtained different results and reported sensitivity, specificity, and accuracy values, respectively, of 52.1%–71.3%, 80%–93.3%, and 64%–71.7% for APW and of 55.7%–81.4%, 93.7%–100%, and 57.9%–82% for RPW. According to these authors, anticipating the acquisition of delayed scans would not provide sufficient time for the wash-out of the adenomas to be completed; so, the scans at 10 minutes would not be effective in clinical practice [20]. Even regarding the wash-out estimated at 5′, there are conflicting opinions in the literature. Kamiyama et al. and Foti et al. reported accuracy values greater than 90% [19, 21], while Taffel et al., in a recent review, suggested that the further reduction of the acquisition time of late scans at 5 minutes would decrease significantly the sensitivity of the test, limiting the clinical value [3].

To our knowledge, in a single paper already reported in the literature, washout curves evaluated by late scans acquired at intervals of 5′, 10′, 15′, 30′, and 45′ were compared, and the authors concluded that a significantly more rapid wash-out for adenomas, compared to nonadenomas, was already evident at 5′, but the authors suggested the use of scans at 15′, being associated with higher sensitivity and specificity values for differential diagnosis (88%–96% for a 60% APW and 96%–100% for a 40% RPW), although no hypothesis in support of the proposed method was reported in this research [12].

Our results confirm the importance of wash-out in the differentiation between adenomas and nonadenomas, with sensitivity, specificity, and diagnostic accuracy values of 96%, 95%, and 96%, respectively. As proposed by Korobkin et al., our results have been obtained by considering APW of more than 60% and RPW of more than 40% as significant for adenomas [12].

In this field, other authors described the possibility of modifying APW and RPW threshold values based on the scan delay after intravenous injection of contrast material. In fact, in a recent series, Park et al. proposed APW values of 35% at 5′, 45% at 10′, and 55%–60% at 15′ for differential diagnosis. These authors reported higher sensitivity and specificity values at 15′, with an APW threshold value of 55% [29]. These data seem different from those obtained in our experience; therefore, this issue cannot be considered as resolved, and further studies are needed in this field. 

The comparison of the wash-out calculated in CT scans at 5′ and 10′ (group 1) and at 5′ and 15′ (group 2) showed small variations of the obtained values and not significant changes for diagnostic accuracy, with high correlation between the APW and RPW.

In particular, in the first group of patients, the mean value of APW for adenomas was equal to 69.8% at 5′ and 75.9% at 10′. For nonadenomas, it was 25.1% at 5′ and 33.5% at 10′. The mean value of RPW for adenomas was 67.2% at 5′ and 73.5% at 10′, while for nonadenomas, it was 15.8% at 5′ and 20.5% at 10′.

In the second group of patients, by considering scans performed at 5′ and 15′, mean values of APW of 63% at 5′ and 73.8% at 15′ and mean values of RPW of 54.6% at 5′ and 65.5% at 15′ emerged for adenomas. In case of nonadenomas, mean values of APW and RPW were, respectively, equal to 22% and 12.5% at 5′ and to 35.5% and 19.9% at 15′. 

In our experience, the densitometric changes useful for differential diagnosis, therefore, were already evident at 5′ and did not significantly change at 10′ and 15′. Even in the case of adrenal metastasis misdiagnosed as adenoma, values of absolute and relative wash-out significant for adenoma were observed both in the scans at 5′ and 10′, as also in the case of the unrecognized adenoma, in which densitometric values significant for nonadenoma were detected both in the scans performed at 5′ and 15′. It should be emphasized that in these two patients, neither unenhanced CT scans were significant for a correct diagnosis. The possibility that nonadenomatous lesions can mimic contrastographic features of adenomas is described in the literature especially in case of pheochromocytomas, which in the series reported by Park et al. showed an APW of more than 60% in delayed scans in 16% of cases [30].

Our research has important limitations essentially represented by the small number of considered patients, and, anyway, it does not contribute to the knowledge regarding the pathophysiology of the more rapid wash-out for adenomas than for nonadenomas. Our results, however, seem to confirm the hypothesis that such behavior is determined by an alteration of capillary permeability in case of nonadenomas, responsible for a prolonged intralesional persistence of contrast material [13]. In case of adenomas, capillary permeability is instead not changed, and the washout is rapid and therefore already evident at 5′ and does not progress significantly in later CT scans.

## 5. Conclusions

Multidetector computed tomography represents an extremely sensitive imaging tool for recognizing adrenal expansive lesions, being able to detect lesions of a few millimeters in diameter.

The intralesional washout is a very accurate parameter for differential diagnosis between adenomas and nonadenomas and is essential in the characterization of adenomas without intracytoplasmic lipids.

The evaluation of the wash-out obtained in CT scans performed at 5′, 10′, and 15′ after the intravenous injection of contrast material provides diagnostic comparable results. CT scans performed at 5′ are, therefore, to be preferred, since they reduce the examination time and patient discomfort.

## Figures and Tables

**Table 1:** Mean densitometric values obtained in group 1.

Group 1		
	Adenomas	Nonadenomas
Mean density in unenhanced phase (HU)	3	31,4
Mean density in portal phase (HU)	76,8	85,9
Mean density in 5′ delayed phase (HU)	24,8	66,1
Mean density in 10′ delayed phase (HU)	21	61,3
5′ mean APW	69,80%	25,10%
5′ mean RPW	67,20%	15,80%
10′ mean APW	75,90%	33,50%
10′ mean RPW	73,51%	20,50%

**Table 2:** Mean densitometric values obtained in group 2.

Group 2		
	Adenomas	Nonadenomas
Mean density in unenhanced phase (HU)	5,27	27,1
Mean density in portal phase (HU)	80,2	61,3
Mean density in 5′ delayed phase (HU)	32,58	53,66
Mean density in 10′ delayed phase (HU)	24,35	49,11
5′ mean APW	63,06%	22,05%
5′ mean RPW	54,65%	12,51%
15′ mean APW	73,81%	35,50%
15′ mean RPW	65,57%	19,95%
